# Prevalence, socio-demographic characteristics, and comorbid health conditions in pre-dialysis chronic kidney disease: results from the Manitoba chronic kidney disease cohort

**DOI:** 10.1186/s12882-018-1058-3

**Published:** 2018-10-10

**Authors:** Mariette J Chartier, Navdeep Tangri, Paul Komenda, Randy Walld, Ina Koseva, Charles Burchill, Kari-Lynne McGowan, Allison Dart

**Affiliations:** 1 Manitoba Centre for Health Policy, Department of Community Health Sciences, University of Manitoba, Winnipeg, Canada; 2 Chronic Disease Innovation Centre, Seven Oaks General Hospital, Department of Medicine and Community Health Sciences, Max Rady College of Medicine, University of Manitoba, Winnipeg, Canada; 3 Department of Pediatrics and Child Health, Section of Nephrology, University of Manitoba, Winnipeg, Canada

**Keywords:** Chronic kidney disease, Prevalence, Comorbidity, Epidemiology, Cohort, Administrative data, Surveillance

## Abstract

**Background:**

Chronic Kidney Disease (CKD) is common and its prevalence has increased steadily over several decades. Monitoring of rates and severity of CKD across populations is critical for policy development and resource planning. Administrative health data alone has insufficient sensitivity for this purpose, therefore utilizing population level laboratory data and novel methodology is required for population-based surveillance. The aims of this study include a) develop the Manitoba CKD Cohort, b) estimate CKD prevalence, c) identify individuals at high risk of progression to kidney failure and d) determine rates of comorbid health conditions.

**Methods:**

Administrative health and laboratory data from April 1996 to March 2012 were linked from the data repository at the Manitoba Centre for Health Policy. Prevalence was estimated using three methods: a) all CKD cases in administrative and laboratory databases; b) all CKD cases captured only through the laboratory data; c) and the capture-recapture method. Patients were stratified by risk by estimated Glomerular Filtration Rate (eGFR) and albuminuria based on Kidney Disease Improving Global Outcomes (KDIGO) criteria. For comorbid health conditions, the counts were modelled using a Generalized Linear Model (GLM).

**Results:**

The Manitoba CKD Cohort consisted of 55,876 people with CKD. Of these, 18,342 were identified using administrative health data, 27,393 with laboratory data, and 10,141 people were identified in both databases. The CKD prevalence was 5.6% using the standard definition, 10.6% using only people captured by the laboratory data and 10.6% using the capture-recapture method. Of the identified cases, 46% were at high risk of progression to end-stage kidney disease (ESKD), 41% were at low risk and 13% were not classified, due to unavailable laboratory data. High risk cases had a higher burden of comorbid conditions.

**Conclusion:**

This study reports a novel methodology for population based CKD surveillance utilizing a combination of administrative health and laboratory data. High rates of CKD at risk of progression to ESKD have been identified with this approach. Given the high rates of comorbidity and associated healthcare costs, these data can be used to develop a targeted and comprehensive public health surveillance strategy that encompass a range of interrelated chronic diseases.

**Electronic supplementary material:**

The online version of this article (10.1186/s12882-018-1058-3) contains supplementary material, which is available to authorized users.

## Background

Chronic kidney disease (CKD) is a common disorder requiring a public health surveillance strategy to identify those at risk of progression and treat them with disease modifying therapies [1]. Epidemiological studies report that CKD is highly prevalent in the general population and that it has increased steadily over several decades [2–4]. Not surprisingly, parallel to these increases in CKD, increases in the prevalence of kidney failure, or End Stage Kidney Disease (ESKD), have also been reported [5, 6]. ESKD is associated with comorbid health conditions, poor quality of life, and high health care costs [7].

It is critical to develop innovative methods to estimate CKD prevalence and identify affected individuals requiring treatment. Kidney failure affects more Manitobans per capita than most other provinces in Canada, with a prevalence rate of 1530 per million population compared to the Canadian rate of 1193 per million population [5]. The under-recognition and treatment of CKD in its earlier stages and the increasing prevalence of risk factors associated with CKD are implicated in high ESKD rates [8]. Appropriate screening for, monitoring and treatment of certain types of CKD can prevent or delay progression to ESKD. Understanding the geographic variations in prevalence informs health services planning.

Unfortunately, information about the incidence and prevalence of earlier stages of CKD in the general population is limited. Estimates of CKD prevalence in Canada range from 10 to 15%, representing approximately 3 million affected adults [2]. Prevalence rates of CKD across Europe, Asia, North America and Australia range between 2.5 and 11.2% [3] and are highest among low to middle income countries [4]. ESKD represents a small fraction of the total CKD population, with early CKD affecting 50 times more individuals than ESKD [6]. Determining patients who are at greater risk of progression to ESKD is an important aspect of CKD surveillance.

In this study, we propose a novel methodologic approach by combining both laboratory and administrative health data to monitor CKD prevalence. Combining these types of data with novel methods to calculate prevalence could overcome the challenges of the low sensitivity of administrative data. The aims of this study are to: a) develop the Manitoba CKD cohort from a wide range of administrative health databases including medical claims, hospitalization records, prescriptions and laboratory data; b) estimate the CKD prevalence in the province of Manitoba using all sources of data and a capture-recapture method; c) determine the percentage of cases who are at highest risk for progression to ESKD; and d) determine rates of comorbid health conditions among people identified in the CKD cohort.

## Methods

### Setting and design

This study was conducted in the Canadian province of Manitoba, with a population of about 1.2 million inhabitants. The majority of the population (59%) live in the capital city of Winnipeg, 36% live in rural southern regions and 5% in the northern region located above the 52 parallel. Given the high prevalence rates of dialysis patients in the province, Manitoba Health requested a comprehensive CKD study. Researchers from the Manitoba Centre for Health Policy (MCHP) and the Manitoba Renal Program collaborated on creating the Manitoba CKD Cohort. This focus included Manitoba adults who were 18 years old and over. A separate pediatric cohort was also created and will be described in a separate paper. The study was approved by the Health Research Ethics Board (HREB) at the University of Manitoba (#H2012:297). Obtaining individual consent for use of administrative health databases has been waived by HREB.

The Manitoba CKD Cohort is based on data collected from the publically funded health care system housed at MCHP. These health services datasets include person-level health records virtually capturing the entire population (> 99%). All records within these datasets are de-identified, and personal health identifiers such as Personal Health Identification Numbers (PHINs) are scrambled [9–11]. The data are linkable across files and over time because the PHIN is scrambled in the same way for each dataset. The following datasets were used in this study: Manitoba Health Insurance Registry, Medical Claims/Medical Services, Physician Registry, Hospital Abstracts, Drug Program Information Network, Diagnostic Services Manitoba Laboratory Data, Vital Statistics and Canada Census Files. Detailed information about these data is available on the MCHP website [12].

### CKD definition using administrative data

The Manitoba CKD cohort included adults living in Manitoba as of March 31, 2012 who met the definition for CKD at some point between April 1, 1996 and March 31, 2012. The CKD cohort was defined using a combination of administrative health and laboratory data. Within the administrative health data, CKD was defined as having at least two CKD-related medical claims by physician visit *or* one CKD-related hospitalization *or* one filled prescription of a medication specifically used in the treatment or management of CKD within a three-year period [13]. To ensure that acute kidney disease was not counted as CKD, ICD codes related to acute kidney disease were excluded and two abnormal blood tests at least 3 months apart were required to count as a CKD case. The medications listed are those used to treat CKD-related anemia, hyperkalemia, elevated phosphate and hyperparathyroidism. Details of the definitions are found in Table 1.

**Table 1:** Chronic kidney disease definitions and codes

Indicators	Definitions and Codes
Chronic kidney disease (using administrative data)	Defined as an adult with the following diagnoses from physician claims and hospital records, and drug prescriptions: • Two or more physician claims with diagnoses for hypertensive chronic kidney disease, hypertensive heart and chronic kidney disease, acute glomerulonephritis, nephrotic syndrome, chronic glomerulonephritis, nephritis and nephropathy not specified as acute or chronic, chronic kidney disease, renal failure (unspecified), renal sclerosis (unspecified), disorders resulting from impaired renal function, hydronephrosis, other disorders of kidney and ureter, congenital anomalies of urinary system (ICD-9-CM: 403, 404, 580, 581, 582, 583, 585, 586, 587, 588, 591, 593, 753), or • One or more hospital episodes with diagnoses for diabetes with renal manifestations, hypertensive chronic kidney disease, hypertensive heart and chronic kidney disease, acute glomerulonephritis, nephrotic syndrome, chronic glomerulonephritis, nephritis and nephropathy not specified as acute or chronic, chronic kidney disease, renal failure (unspecified), renal sclerosis (unspecified), disorders resulting from impaired renal function, hydronephrosis, unspecified disorder of kidney and ureter, congenital anomalies of urinary system (ICD-9-CM: 250.4, 403, 404, 580, 581, 582, 583, 585,586, 587, 588, 591, 593.9, 753; ICD-10-CA: E10.2, E11.2, I12, I13, N18, N19, N00–16, N25, N26, N28.82, N39.1, Q60–64), or • One or more filled drug prescriptions used in CKD management: ○ generic names: Epoetin Alfa, Darbepoetin Alfa, Peginesatide, Sodium Polystyrene Sulfonate, Calcium Polystyrene sulphonate, Polystyrene Sod Sulfonate 454, Sevelamer HCL, Cinacalcet HCL, Lanthanum; ○ Anatomical Therapeutic Chemical (ATC) codes: B03XA01, B03XA02, B03XA04, V03AE01, V03AE02, H05BX01, V03AE03.
Chronic kidney disease (using laboratory data)	Defined as an adult with: 1) Two abnormal estimated Glomerular Filtration Rate (eGFR) tests at least 90 days apart using the following equation: Modification of Diet in Renal Disease (MDRD) equation [14] Men: *(175 x (S*_*cr*_*/88.4)*^*-1.154*^*)) x (AGE)*^*-0.203*^*)* Women: multiply results by 0.74). OR 2) Two abnormal tests for proteinuria (either Protein-Creatinine Ratio (PCR), Albumin-Creatinine Ratio (ACR) or dipstick protein urinalysis) at least 90 days apart. Abnormal test values were defined as: • eGFR values < 60 ml/min/1.73m^2^ • PCR > =15 mg/mmol or ACR > =3 mg/mmol [14] • Dipstick Protein Urinalysis gives a categorical measurement of urine protein (0 to 4) which is less precise than a Urine Protein test*. A dipstick protein level greater than or equal to 0.3 g/L is considered abnormally high. * Note: A comparison of same-day Dipstick and Urine Protein test results showed moderate agreement (kappa = 0.48): • Dipstick Protein = 0 comparable to Urine Protein < 15 mg/mmol • Dipstick Protein = 1 comparable to Urine Protein 15–50 mg/mmol • Dipstick Protein = 2–4 comparable to Urine Protein > 50 mg/mmol

### CKD definition using laboratory data

For people with laboratory results, we defined CKD by estimating the Glomerular Filtration Rate (eGFR) and level of proteinuria. We determined that an adult had CKD if he/she had at least two tests indicating eGFR < 60 ml/min/1.73m^2^ at least 90 days apart as shown in Table 1. The Modification of Diet in Renal Disease (MDRD) equation was chosen to estimate the eGFR because it was being used locally by the provincial laboratories. Proteinuria was assessed by urine protein to creatinine ratio (PCR), urine albumin to creatinine ratio (ACR) or dipstick protein urinalysis. As shown in Table 1, we compared dipstick and urine protein results and oufnd a moderate agreement (kappa = 0.48). We ensured that the two tests were at least 90 days apart to address concerns of one-off testing which may lead to overestimation of CKD cases [15]. Tests with missing date of collection were excluded.

### Risk of progression to ESKD

**Fig. 1:**
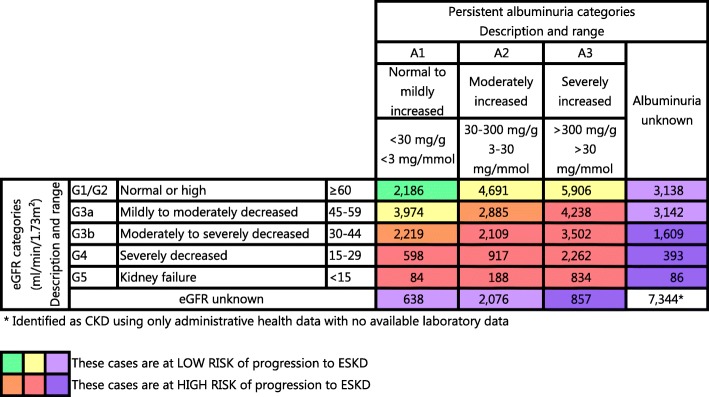
Heat map of adults with chronic kidney disease by risk of progression to ESKD

As shown in Fig. 1, we adapted a heat map, colour-coded classification, developed by the Kidney Disease Improving Global Outcomes (KDIGO) Work Group [14]. The heat map incorporated albuminuria levels, given that albuminuria is an important marker of kidney function and risk of progression to ESKD. We categorized the CKD cases by risk of progression to ESKD, using the eGFR and albuminuria levels to identify four levels of risk: a) lowest risk (green), b) moderately increased risk (yellow); c) high risk (orange) and d) the highest risk of all (red).

We adapted the KDIGO heat map by adding an additional category as shown by the light and dark purple boxes, because we wanted to use the available laboratory data to categorize as many cases as possible. Some CKD cases identified through the administrative health data had some laboratory data, but were missing either the creatinine or albuminuria. These cases could be categorized as low risk (light purple) or high risk (dark purple). CKD cases with no laboratory data were categorized as “unknown risk” given that no information about kidney function impairment was available. These classifications were then combined into a low risk (green, yellow, and light purple) and high risk (orange, red, and dark purple). This classification permitted us to examine these CDK groups: the low-risk group that includes earlier stages of CKD, the high-risk group that includes later stages of CKD, unknown CKD group, where the risk to progression to ESKD was unknown.

### Sociodemographic characteristics and health conditions

We estimated the CKD prevalence by age and sex, as obtained in the health registry data files, and by income quintiles. The income quintiles were based on the average household income of small geographical areas (made up of 400 to 700 people) from the 2006 and 2011 Canadian census data, and ranking incomes in these areas from lowest to highest. Indicators for the following health conditions were defined utilizing the physician claims, hospital records and prescription data: diabetes, lower-limb amputation (among those with diabetes), hypertension, ischemic health disease, congestive heart failure, acute myocardial infarction, stroke, and atrial fibrillation. The codes for these definitions are found in the Additional file 1: Table S1 entitled, *Definitions of Comorbid Health Conditions*.

### Statistical analyses

CKD prevalence was estimated using three methods. The first consisted of counting all CKD cases in administrative health and laboratory databases and dividing by the total Manitoba population. The second used exclusively laboratory data and divided CKD cases by all persons with laboratory data required to make a CKD diagnosis. For the third estimate, we used the following capture-recapture method or Chapman formula [16]:

*((number of cases in administrative health data + 1) x (number of cases in laboratory data + 1) / (number of cases in both + 1)) -1.*

The capture-recapture methodology is a novel approach to estimating disease prevalence, a method originally used to determine the size of animal populations [17, 18]. This method requires databases of cases from two different but potentially overlapping sources, thus taking advantage of the administrative data and the laboratory data from the Manitoba population. The idea is that disease prevalence will be higher in populations where there are fewer overlapping cases from two separate data sources. The standard practice of simply merging several data sources will likely miss cases and underestimate the disease prevalence [19].

Generalized Linear Model (GLM) was used estimate the prevalence of comorbid health conditions in the CKD cohort as it is suitable for non–normally distributed data such as indicator counts. Depending on which fit the data best, various distributions were used for different indicators, including Poisson distribution, negative binomial distribution, or binomial distribution. Rates were age and sex adjusted. SAS® version 9.3 software was used to conduct all data management, programming and analyses on MCHP’s secure server.

## Results

**Fig. 2:**
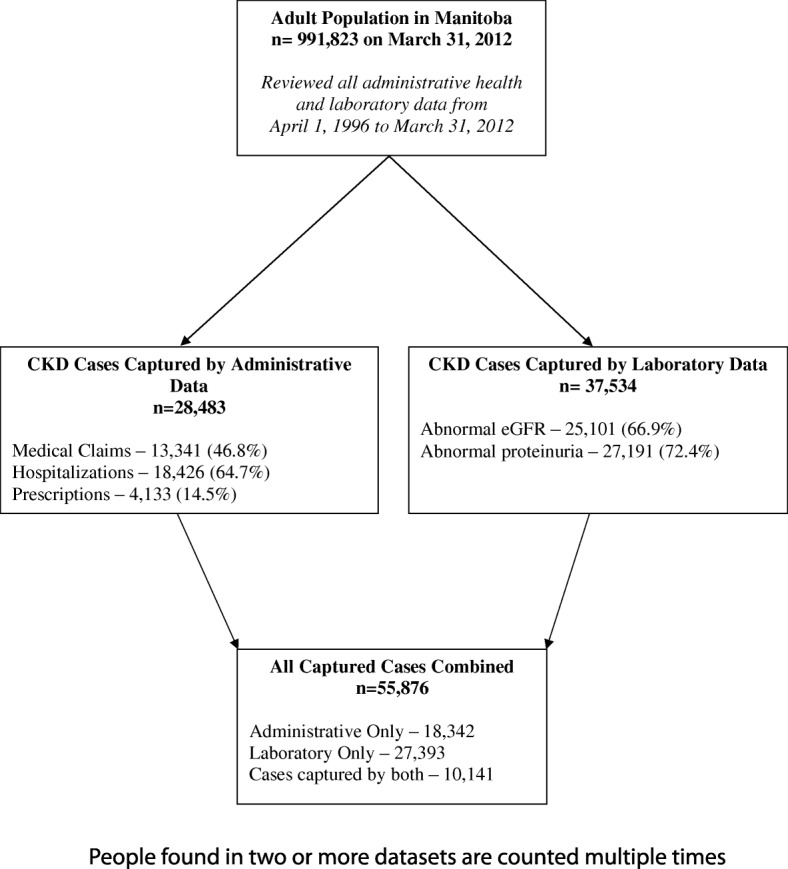
Flow Chart for creating the Manitoba chronic kidney disease cohort

Figure 2 illustrates how the cohort was created. The total number of Manitoba adults (18 years and older) with CKD found in all databases was 55,876. Of these, 18,342 were identified using administrative health data, 27,393 were found in the laboratory data, and 10,141 people were found in both databases. Table 2 shows the characteristics of the Manitoba CKD Cohort (*n* = 55, 876). We note that these characteristics differ from the total Manitoba population (*n* = 991,823). More women than men were captured in the CKD cohort. Over half of CKD cases were 65 years and older. When comparing the percentages of people residing in the rural health regions and the main urban health region, we note that CKD cases in some rural regions appear to be underrepresented.

**Table 2:** Chronic kidney disease cohort and total Manitoba population characteristics

Characteristics	CKD Cohort Counts (%) *n* = 55,876	Manitoba 2012 Population Counts (%) *n* = 991,823
Sex		
Men	24,947 (44.6%)	485,948 (49.0%)
Women	30,929 (55.4%)	505,875 (51.0%)
Age Groups		
18–44 years old	9681 (17.3%)	469,102 (47.3%)
45 to 64 years old	15,750 (28.2%)	338,726 (34.2%)
65 and older	30,445 (54.5%)	183,995 (49.0%)
Income Quintiles		
Rural 1 (lowest)	3614 (6.5%)	66,555 (6.7%)
Rural 2	3203 (5.7%)	74,715 (7.5%)
Rural 3	3322 (5.9%)	76,648 (7.7%)
Rural 4	3270 (5.9%)	71,984 (7.3%)
Rural 5 (highest)	2828 (5.1%)	74,853 (7.5%)
Urban 1 (lowest)	9593 (17.2%)	121,379 (12.2%)
Urban 2	8213 (14.7%)	125,383 (12.6%)
Urban 3	7213 (12.9%)	123,252 (12.4%)
Urban 4	6414 (11.5%)	123,541 (12.5%)
Urban 5 (highest)	6051 (10.8%)	124,367 (12.5%)
Health Regions		
Southern Health (rural)	4649 (8.3%)	133,549 (13.5%)
Winnipeg (urban)	38,055 (68.1%)	582,923 (58.8%)
Prairie Mountain Health (rural)	4918 (8.8%)	129,480 (13.1%)
Interlake-Eastern (rural)	5468 (9.8%)	97,375 (9.8%)
Northern (rural)	2782 (5.0%)	48,491 (4.9%)
Remote Communities*	1925 (3.4%)	18,077 (1.8%)

* These are remote northern communities that do not have permanent road access, are more than a four-hour drive from a major rural hospital or have rail or fly-in access only. These communities are found in Interlake-Eastern and Northern Health Regions, therefore the numbers in these remote communities overlap with the numbers of these health regions

### CKD prevalence

The prevalence among all adults with CKD identified in both administrative and laboratory data was 5.6% (*n* = 55,876). Among adults in Manitoba with laboratory data, the CKD prevalence was 10.6% (*n* = 37,534). Prevalence based on the capture-recapture method, which uses cases defined both by administrative and laboratory data, also reached 10.6% (*n* = 105,417). These estimates exclude ESKD cases (*n* = 1854) on dialysis in the Manitoba Renal Program and with kidney transplants.

Figure 1 shows the CKD cohort categorized by level of risk of progression to ESKD based on available laboratory data. Of the 55,876 people with CKD, approximately 41% (*n* = 23,037) were at low risk (green, yellow, and light purple). About 46% (*n* = 25,495) had more advanced CKD and were at higher risk of progression. The CKD stage and risk level were unknown for 13% (*n* = 7344) due to unavailable laboratory data.

### Socio-demographic characteristics

Rates of CKD were higher in women. For women, 31.8/1000 were found to be have CKD with high risk of progression to ESKD versus 25.9/1000 for men. Higher rates of all categories of CKD were found among older compared to younger populations. Rates of high risk CKD among adults aged 65 years and older were 97.9/1000, adults aged 45 to 64 years, 20.6/1000, and adults aged 18 to 44 years, 7.9/1000 (Table 3).

**Table 3:** CKD prevalence by sex, age and risk of progression. Crude rates per 1000 adults as of March 31, 2012

Indicators	CKD Risk of Progression		
	Unknown	Low	High
Sex			
Males	6.9	18.6	25.9
Females*	7.9	21.4	31.8
Age (years)			
18–44	4.3	8.5	7.9
45–64*	6.4	19.6	20.6
65 and older*	17.3	50.3	97.9

* Indicates all categories of risk to progression were statistically different from the reference group (age: 18–44 or Sex: Male)

**Fig. 3:**
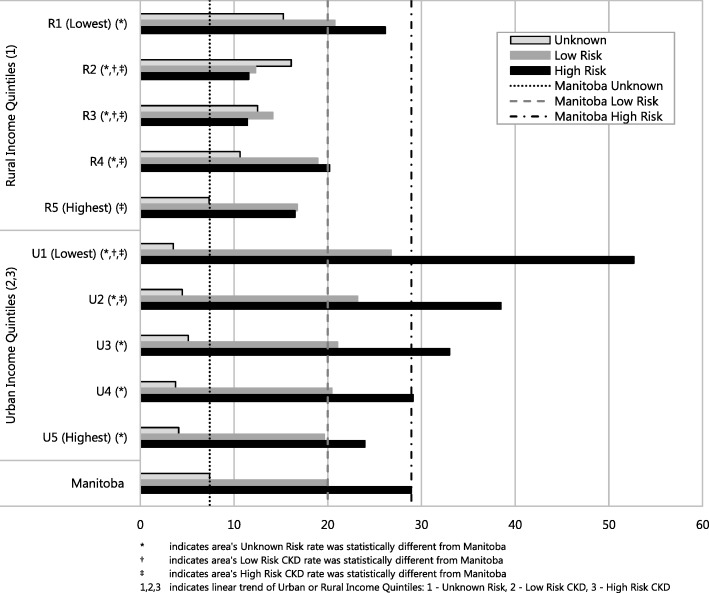
Chronic kidney disease prevalence by rural and urban income quintiles

In urban areas, with the exception of the group of unknown risk, there was a linear trend across income quintiles, meaning that as income increased, a lower prevalence of CKD was found (Fig. 3). The converse was found in the rural areas, where this linear trend was found only in the group of unknown risk. The different patterns between urban and rural areas are likely due to incomplete coverage of laboratory data in rural areas.

**Fig. 4:**
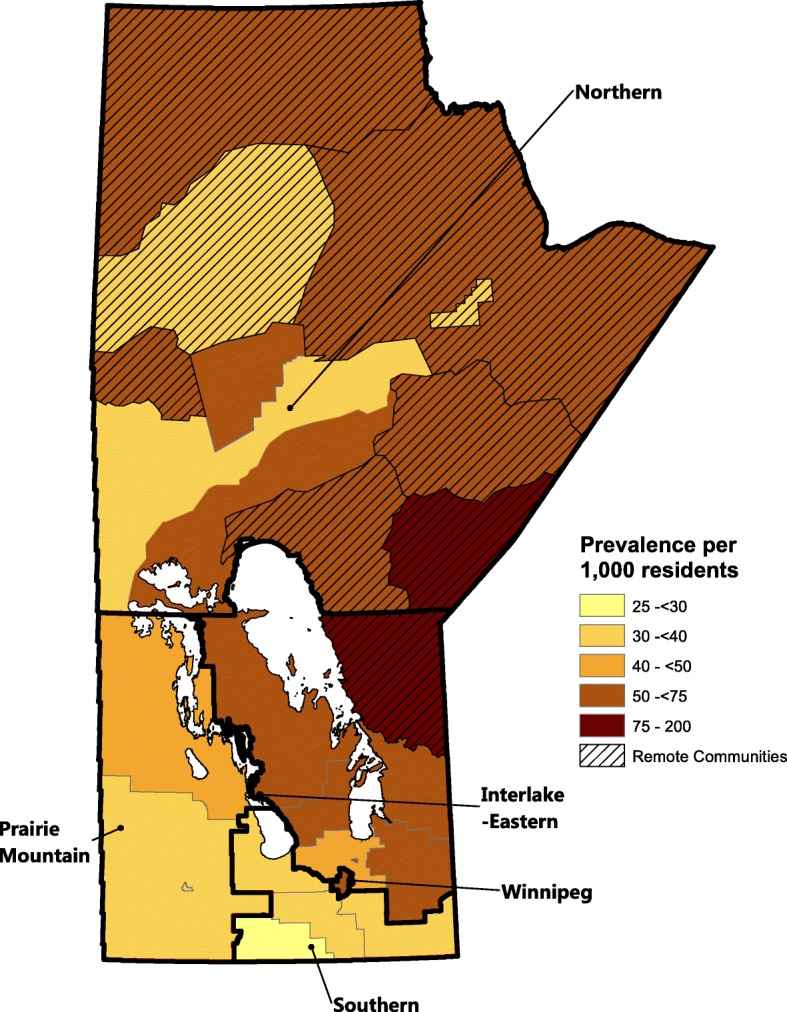
Chronic kidney disease prevalence by Manitoba health regions

We also noted important differences in CKD prevalence across health regions in Manitoba as shown in Fig. 4. The most northern area in the province, particularly in the remote areas (denoted by the diagonal lines), has consistently high rates. The South Eastern region of the province has the lowest CKD rates of the province.

### Comorbid health conditions

The overall burden of comorbid medical conditions was considerably higher among people with CKD than among those without CKD (Table 4). For example, the prevalence of ischemic heart disease among the high risk CKD group was 24.55% compared to 4.72% in the No CKD group. Rates of comorbid medical conditions also increased with severity of CKD. For example, relative to those without CKD, rates of hypertension were higher among people classified with CKD at lower risk (RR: 2.24) and higher still among people with CKD at higher risk (RR: 3.02). Similarly, rates of diabetes and lower-limb amputation were higher among all groups of CKD compared to those without CKD, but highest among people with high risk of progressing to ESKD. While the unknown risk group has lower rates of comorbid health conditions compared with the low-risk and high-risk groups, they have statistically higher rates of several of the conditions including diabetes, amputation, congestive heart failure, stroke and atrial fibrillation.

**Table 4:** Comorbid health conditions among adults with chronic kidney disease by risk of progression. Age and sex adjusted prevalence and relative risks, 95% confidence intervals

Indicators		No CKD *N* = 935,947	CKD by Risk of Progression to ESKD		
			Unknown *n* = 7344	Low *n* = 23,037	High *n* = 25,495
Diabetes (%) (2009/10–2011/12)	Prevalence	7.02 (6.96–7.07)	12.44 (8.64–17.92)	28.59 (19.80–41.30)	37.10 (25.62–53.73)
	Relative Risk	reference	**1.77 (1.23–2.55)**	**4.08 (2.82–5.89)**	**5.29 (3.65–7.66)**
Hypertension (%) (2011/12)	Prevalence	21.45 (21.36–21.55)	31.06 (19.91–48.45)	43.84 (28.01–68.61)	61.46 (39.06–96.72)
	Relative Risk	reference	1.45 (0.93–2.26)	**2.04 (1.31–3.20)**	**2.87 (1.82–4.51)**
Ischemic Heart Disease (%) (2007/08–2011/12)	Prevalence	4.72 (4.68–4.77)	7.30 (4.29–12.43)	12.22 (7.20–20.74)	24.55 (14.25–42.27)
	Relative Risk	reference	1.55 (0.91–2.63)	**2.59 (1.52–4.39)**	**5.20 (3.02–8.95)**
Acute Myocardial Infarction (%) (2007/08–2011/12)	Prevalence	0.99 (0.96–1.01)	1.10 (0.63–1.93)	2.29 (1.37–3.81)	5.29 (3.12–8.97)
	Relative Risk	reference	1.12 (0.64–1.96)	**2.32 (1.40–3.87)**	**5.37 (3.16–9.11)**
Congestive Heart Failure (%) (2009/10–2011/12)	Prevalence	1.38 (1.35–1.41)	4.51 (2.47–8.26)	5.19 (2.88–9.35)	25.09 (13.64–46.17)
	Relative Risk	reference	**3.27 (1.79–5.98)**	**3.76 (2.09–6.77)**	**18.17 (9.88–33.43)**
Stroke (%) (2007/08–2011/12)	Prevalence	0.41 (0.39–0.43)	0.92 (0.51–1.67)	2.01 (1.17–3.45)	5.59 (3.13–9.98)
	Relative Risk	reference	**2.26 (1.25–4.10)**	**4.94 (2.88–8.47)**	**13.72 (7.68–24.50)**
Atrial Fibrillation (%) (2009/10–2011/12)	Prevalence	1.92 (1.89–1.94)	3.09 (2.20–4.33)	5.03 (3.64–6.94)	9.72 (6.97–13.57)
	Relative Risk	reference	**1.61 (1.15–2.26)**	**2.63 (1.90–3.63)**	**5.08 (3.64–7.09)**
Lower-Limb Amputation Among Diabetics (%) (2007/08–2011/12)	Prevalence	0.33 (0.29–0.38)	0.79 (0.44–1.42)	0.77 (0.52–1.13)	2.65 (1.83–3.84)
	Relative Risk	reference	**2.37 (1.32–4.27)**	**2.30 (1.55–3.42)**	**8.00 (5.53–11.57)**

**Bolded** values indicate statistically significant difference from the No CKD group

#### Risk factors associated with CKD

Table 5 shows that the highest percentages of CKD cases were found among adults older than 65 and with hypertension and diabetes. This suggests that the Manitoba CKD Cohort is capturing adults at greatest risk of developing CKD. The unknown cases were identified through the administrative health data, but no lab data was available to categorize them by low risk or high risk. It is reassuring to see that a greater percentage of lab tests were available on the groups with hypertension, diabetes and older age than the group with no risk factors.

**Table 5:** The percentage and number* of adults at risk of progression to ESKD among those with and without risk factors for ESKD, March 31, 2012

Risk of Progression ESKD	No risk factors (*N* = 672,495)	Hypertension (*N* = 232,797)	Diabetes (*N* = 84,405)	Over 65 (*N* = 183,375)
	% (n)	% (n)	% (n)	% (n)
No CKD (no risk)	98.4% (661,404)	83.5% (194,447)	75.7% (63,907)	83.4%(152,930)
CKD Unknown Risk	0.4% (2905)	1.6% (3661)	1.7% (1394)	1.7% (3188)
CKD Low Risk	1.0% (6751)	6.7% (15,629)	11.0% (9278)	5.9% (10,783)
CKD High Risk	0.2% (1435)	8.2% (19,060)	11.6% (9826)	9.0% (16,474)

*The numbers to not add up to the total population because there is overlap between the risk factors

## Discussion

This study describes the development of the Manitoba CKD Cohort using a combination of administrative health and laboratory data, as well as how adult CKD prevalence was estimated. We found that the CKD prevalence was 5.6%, using the standard method and 10.6% using only people with laboratory data. Using the capture-recapture method, CKD prevalence was also estimated at 10.6% which is in line with previous studies [2]. Of the identified adult CKD cases, 46% were at high risk of progression to ESKD and 41% were at low risk. The remaining 13% did not have any laboratory data and were therefore not categorized by risk. This study also found the expected high rates of comorbid health conditions among adults with CKD, which increased from the low to the high risk cohort, supporting the methodology utilized to assess cases.

The estimated prevalence of 10.6% found in two of the methods used supports the face validity of our approach, although given that ESKD rates in Manitoba are amongst the highest in Canada, these may be underestimates. In a national study, using the Canadian Health Measures survey, Arora et al. [2] found that the prevalence of CKD in Canadian adults ranged from 10 to 15%. Similarly, other investigators, reported CKD rates ranging between 2.5 and 11% across Europe, Asia and North America, and found CKD rates ranging from 4.7 to 33% in low- to middle-income countries. Our results suggest that CKD rates in Manitoba may be at the higher end of the range for high-income countries.

The burden of other health conditions in our CKD cohort is much higher than the provincial average for all the indicators we examined [20]. In the high-risk CKD group alone, the rate of stroke was over 14 times higher than the Manitoba average in 2011/12, and the prevalence of diabetes was over five times higher. Previous studies have found that comorbidities are common in CKD, even in the early stages of the disease and that these comorbidities are associated with increased treatment burden and poorer quality of life [21]. In addition, we found a trend of increasing comorbidity with increasing risk of progression. Previous research shows that rates of comorbid diseases at the CKD stages 3–5 are double the rates at stages 1–2 [2, 22]. Go, Chertow, Fan et al., [23] in a large epidemiologic study, found a graded association between a reduced eGFR and the risk of death and cardiovascular events. A meta-analysis reported that the risk of myocardial infarction increased with lower eGFRs [24].

Our study enabled us to capture individuals through the health care system, who may be difficult to reach in screening/surveillance studies. This allowed us to evaluate the geographical distribution and social factors associated with CKD. Our findings suggest that CKD is not equally distributed across geographic regions and socioeconomic status. The CKD rates increase with decreases in income. We also observed that the highest rates of CKD in remote communities. These remote communities are largely populated by Indigenous peoples where living conditions are challenging due to economic conditions, poor water supply and access to affordable food, as well as limited health, social and recreational services. These findings are consistent with previous research. A recent review found that socially disadvantaged CKD patients had poorer access to health services, and higher rates of cardiovascular events and mortality than more advantaged patients [25]. Despite Canada’s universal healthcare system, people living in poverty and in remote communities, face barriers to accessing early intervention strategies and treatments that are required to prevent and manage CKD. Improving living conditions and ensuring that economic and social resources are available throughout the province would potentially decrease the development of CKD and other chronic diseases. While there are no simple solutions to reducing poverty, some existing strategies have demonstrated improved outcomes [26, 27].

Estimating CKD prevalence has proven to be challenging because the disease is typically without symptoms in the early stages, general population screening is not currently recommended and vulnerable populations at risk may not present for opportunistic screening even if it is offered. Given that survey respondents might be unaware of indicators of kidney disease, health questionnaires are problematic, and incur considerable cost and effort to collect the information. Remote and isolated regions are often not included in large surveys due to lack of infrastructure such as telephones and roads.

Epidemiological studies have relied on either laboratory testing or administrative health data to estimate population prevalence. Arora and colleagues [2] collected health information as well as laboratory tests required to identify CKD cases, from a representative sample of 3689 respondents across Canada. Laboratory tests to detect CKD including proteinuria and eGFR, can not only determine the presence of CKD, but also the risk of the disease progressing to ESKD. Specifically, by categorizing these tests, individuals can be stratified into low risk, moderately increased risk, high risk, and very high risk for progression to ESKD [14, 28–33]. Administrative databases can be an additional tool to overcome some of the challenges related to cost, time and reaching affected populations. In Alberta for example, a series of definitions for CKD using medical claims and hospitalizations based on the province’s health system were compared to a gold standard [13]. Despite using a longer period, the study found that the definition had 23% sensitivity and 96% specificity. These results indicate that using administrative data to estimate CKD prevalence tends to underreport the true rates and requires validation with population-based laboratory data or large-scale screening initiatives.

In our study, we found that many cases of CKD were not identified through the administrative health data, suggesting that these CKD cases may go undetected and undiagnosed. Given the high prevalence of CKD, it is imperative that primary care physicians conduct routine health checks for at-risk populations. The KDIGO clinical guidelines for CKD [14] and the Canadian Diabetes Association guidelines [34] include recommendations for lifestyle counselling, control of blood sugar and hypertension and avoidance of nephrotoxic substances. Early identification of people at greater risk for progressing to ESKD provides the opportunity for lifestyle counselling to address risk factors and treatments to slow the progression of the disease. Clinical care pathways are available to guide primary care practitioners in the treatment of CKD patients [35]. Furthermore, there is opportunity for increased knowledge translation to ensure that all care practitioners are aware of these guidelines and have access to them in their practices. To ensure that remote communities have access to primary and renal healthcare, alternative models of care should be considered, include increasing recruitment of healthcare providers and using technology to link remote areas to specialized services in larger urban areas. It is important to develop a rigorous evaluation framework to monitor changes in the prevalence of ESKD and high-risk CKD over time in order to increase our understanding of the effectiveness of established screening, surveillance and intervention strategies and shed light on how to improve them.

This study has demonstrated the value of using of innovative methods to estimate CKD prevalence by taking advantage of the overlap between administrative and laboratory datasets. The prevalence estimated by the capture-recapture method is consistent with epidemiologic studies and with the estimate calculated using the laboratory data only. This method has previously been utilized for estimating prevalence of diseases such as acute hepatitis A, diabetes, spina bifida and infants’ congenital anomaly [36]. The prevalence by the capture-recapture method may be an underestimation. Two assumptions are required when using the capture-recapture method: that the dataset includes individual identifiers and that one list does not affect the chance of being on the other list. The second assumption may not be met, because laboratory reports and physician claims are likely related.

### Strengths and limitations

A strength of the Manitoba CKD Cohort is the population-based data sources on which it was created. These data sources included medical claims, hospitalization records, and prescription records that covered virtually all Manitoba residents. It also included laboratory records that covered large portions of the population. The time and costs of linking data is considerably less than collecting large-scale survey data. Previous studies found that CKD rates using administrative health data alone are underestimated, because many CKD cases are not detected by physicians and may not be coded as a diagnosis in the context of other chronic diseases. The addition of laboratory databases addresses some of these limitations by increasing the amount of cases detected. This study shows that using a combination of health and laboratory data provides a more reliable estimate of the CKD prevalence and could be utilized to monitor population-level CKD rates over time.

The availability of the laboratory data permitted us to categorize CKD cases by risk to progression to ESKD. Unfortunately, 13% were of unknown risk because we did not have laboratory data on all CKD cases. Based on the rates of comorbid health conditions in this unknown risk group, we suspect that these cases are in early stages of CKD and being followed in the community by general practitioners.

We acknowledge that relying on prescriptions to determine CKD cases will require further validation. Although, the medications were given careful consideration and are believed to be primarily used in the treatment of CKD, we cannot be certain that all individuals using these medications have CKD. Our rationale for using prescriptions for capturing CKD cases is that using only medical claims and hospitalization records would significantly underestimate the CKD prevalence as shown by Ronksley and colleagues (2012). Even when CKD has been diagnosed, physicians may be coding another co-occurring disorder on the medical claim.

We also recognize possible limitations in how GFR was estimated and challenges with our laboratory data coverage. Measuring GFR accurately requires using multiple blood and urine samples, which are costly and cumbersome [15]. It is important to keep in mind that estimating GFR may be associated with an overestimation. With regards to the laboratory data, these are collected in facilities that provide public laboratory services and diagnostic imaging. However, data from some hospitals, private laboratories and laboratories in Western Manitoba were not captured [37]. Additionally, the geographical distribution of available DSM data was unequal. Whereas 59% of the Manitoba population resides in Winnipeg, 78% of the lab data was from these residents. This resulted in an underestimation of the disease burden in some rural areas. A more accurate picture of CKD in Manitoba will emerge once additional data is added to the Repository, which will be a valuable resource for future studies.

## Conclusions

Chronic kidney disease (CKD) is a health issue of increasing worldwide importance. Utilizing a combination of administrative health and laboratory data, this study found high CKD rates in Manitoba, with a large proportion at risk for progressing to ESKD. Given the high rates of comorbidity, it is important to have a comprehensive public health strategy that encompasses a range of interrelated chronic diseases. Our methodology may be well suited in the creation of passive CKD surveillance systems to target patients who may benefit from early intervention to prevent progression to more advanced forms of CKD and ESKD.

## Additional file

Additional file 1: **Table S1.** Definitions for Comorbid Health Conditions. Provides the diagnostic codes used to define the Comorbid Health Conditions (DOCX 22 kb)

## Abbreviations

ACR: Albumin-creatinine ratio
CKD: Chronic kidney disease
DSM: Diagnostic Services Manitoba
eGFR: estimated Glomerular Filtration Rate
ESKD: End stage kidney disease
KDIGO: Kidney Disease Improving Global Outcomes
MCHP: Manitoba Centre for Health Policy
MDRD: Modification of Diet in Renal Disease
PCR: Protein-creatinine ratio
PHIN: Personal Health Identification Number
RR: Relative risk
SCr: Serum creatinine
