# Supplementary material for: Prevalence, socio-demographic characteristics, and comorbid health conditions in pre-dialysis chronic kidney disease: results from the Manitoba chronic kidney disease cohort
Source: BMC Nephrol. 2018 Oct 10;19:255. doi: 10.1186/s12882-018-1058-3 (PMC6180583; doi:10.1186/s12882-018-1058-3)
Supplement: Supplementary file 1 — Table S1. Definitions for Comorbid Health Conditions. Provides the diagnostic codes used to define the Comorbid Health Conditions (DOCX 22 kb) [file 12882_2018_1058_MOESM1_ESM.docx]

**Table S1: Definitions for Comorbid Health Conditions**

| Health Conditions | Definitions and Codes |
| --- | --- |
| Diabetes | Defined as adults receiving the following diagnoses or prescriptions in the three-year fiscal period 2009/10-2011/12:   - One or more inpatient hospitalizations with a diabetes diagnosis (ICD-9-CM: 250, ICD-10-CA: E10-E14), or - Two or more physician claims with diabetes diagnosis (prefix=7, ICD-9-CM: 250), or - One or more prescription drugs for diabetes treatment (ATC code A10) |
| Lower-limb amputation among those with diabetes | Defined as adults receiving the following diagnoses and procedure or intervention codes in the five-year fiscal period 2007/08-2011/12:   - One or more hospitalizations with a procedure indicating lower-limb amputation (ICD-9: 84.10-84.17, CCI: 1.VC.93, 1.VG.93, 1.VQ.93, 1.WA.93, 1.WE.93, 1.WJ.93, 1.WL.93, 1.WM.93) and a diagnosis for diabetes (ICD-9-CM: 250, ICD-10-CA: E10-E14).   Exclusions:   - Defined only among those meeting the definition for diabetes - Diagnosis code for accidental injury in same hospitalization (ICD-9-CM: 895-897, ICD-10-CA: S78, S88, S98, T05.3, T05.4, T05.5, T13.6) - Interventions coded as out-of-hospital |
| Hypertension | Defined as adults receiving the following diagnoses or prescriptions in the one-year fiscal period 2011/12:   - One or more inpatient hospitalizations for hypertensive disease (ICD-9-CM: 401-405, ICD-10-CA: I10-I13, I15), or - One or more physician claims for hypertensive disease (prefix=7, ICD-9-CM: 401-405), or - One or more prescriptions for antihypertensive drugs, diuretics, beta blocking agents, calcium channel blockers, agents acting on the renin-angiotensin system or terazosin with the following ATC codes: C02AB01, C02AB02, C02AC01, C02CA04, C02CA05, C02DB02, C02DC01, C02LA01, C02LB01, C03AA03, C03BA04, C03BA11, C03CA01, C03CA02, C03CC01, C03DA01, C03DB01, C03DB02, C03EA01, C07AA02, C07AA03, C07AA05, C07AA06, C07AA12, C07AB02, C07AB03, C07AB04, C07AB07, C07AG01, C07BA05, C07BA06, C07CA03, C07CB03, C08CA01, C08CA02, C08CA04, C08CA05, C08CA06, C08DA01, C08DB01, C09AA01, C09AA02, C09AA03, C09AA04, C09AA05, C09AA06, C09AA07, C09AA08, C09AA09, C09AA10, C09BA02, C09BA03, C09BA04, C09BA06, C09BA08, C09BB10, C09CA01, C09CA02, C09CA03, C09CA04, C09CA06, C09CA07, C09CA08, C09DA01, C09DA02, C09DA03, C09DA04, C09DA06, C09DA07, C09DA08, C09DB02, C09XA02, C09XA52, C10BX03, G04CA03   Exclusions:   - Generic name spironolactone - DINs 00028606, 00180408, 00285455, 00594377, 00613215, 00613223, 00613231, 00657182 |
| Ischemic Heart Disease | Defined as adults receiving the following diagnoses or prescriptions in the five-year fiscal period 2007/08-2011/12:   - One or more inpatient hospitalizations for ischemic heart disease (ICD-9-CM: 410-414, ICD-10-CA: I20-I22, I24, I25), or - Two or more physician claims for ischemic heart disease (prefix=7, ICD-9-CM: 401-405), or - One or more prescriptions for platelet aggregation inhibitors, organic nitrates, ubidecarenone, reserpine and diuretics, beta blocking agents, calcium channel blockers, agents acting on the renin-angiotensin system, HMG CoA reductase inhibitors, fibrates, ezetimibe with the following ATC codes: B01AC04, B01AC22, B01AC24, C01DA02, C01DA05, C01DA08, C01DA14, C01EB09, C02LA01, C07AA02, C07AA03, C07AA05, C07AA06, C07AA12, C07AB02, C07AB03, C07AB04, C07AB07, C07AG01, C07BA05, C07BA06, C07BA12, C07CA03, C07CB03, C08CA01, C08CA02, C08CA04, C08CA05, C08CA06, C08DA01, C08DB01, C09AA01, C09AA02, C09AA03, C09AA04, C09AA05, C09AA06, C09AA07, C09AA08, C09AA09, C09AA10, C09BA02, C09BA03, C09BA04, C09BA06, C09BA08, C09CA01, C09CA02, C09CA03, C09CA04, C09CA06, C09CA07, C09CA08, C09DA01, C09DA02, C09DA03, C09DA04, C09DA06, C09DA07, C09DA08, C09DB02, C10AA01, C10AA02, C10AA03, C10AA04, C10AA05, C10AA06, C10AA07, C10AA08, C10AB04, C10AB05, C10AB02, C10AX09, C10BX03, or - One or more prescriptions for low-dose aspirin (≤ 325 mg; DINs: N02BA01, N02BA51, N02BA71) |
| Congestive Heart Failure | Defined as adults receiving the following diagnoses or prescriptions in the three-year fiscal period 2009/10-2011/12:   - One or more inpatient hospitalizations with congestive heart failure (ICD-9-CM: 428, ICD-10-CA: I50), or - Two or more physician claims with congestive heart failure (prefix=7, ICD-9-CM: 428) |
| Acute Myocardial Infarction | Defined as adults receiving the following diagnoses in the five-year fiscal period 2007/08-2011/12:   - One or more hospitalizations with a most responsible diagnosis of AMI and length of stay of three or more days (ICD-9-CM: 410, ICD-10-CA: I21), or - Vital Statistics cause of death coded as AMI (ICD-10: I21) |
| Stroke | Defined as adults receiving the following diagnoses in the five-year fiscal period 2007/08- 2011/12:   - One or more hospitalizations with a most responsible diagnosis of stroke (ICD-9-CM: 431, 434, 436; ICD-10-CA: I61, I63, I64), or - Vital Statistics cause of death coded as stroke (ICD-10: I61, I63, I64) |
| Atrial fibrillation | Defined as adults receiving the following diagnoses or prescriptions in the three-year fiscal period 2009/10- 2011/12:   - One or more inpatient hospitalizations with diagnosis of atrial fibrillation (ICD-9-CM: 427, ICD-10-CA: I48), or - Two or more physician claims with diagnoses for atrial fibrillation (prefix=7, ICD-9-CM: 427) |
